# The effects of *Piper sarmentosum* aqueous extracts on zebrafish (*Danio rerio*) embryos and caudal fin tissue regeneration

**DOI:** 10.1038/s41598-020-70962-7

**Published:** 2020-08-25

**Authors:** Intan Zarina Zainol Abidin, Shazrul Fazry, Nur Hidayah Jamar, Herryawan Ryadi Ediwar Dyari, Zaidah Zainal Ariffin, Anis Nabilah Johari, Nur Suhanawati Ashaari, Nor Azfa Johari, Rohaya Megat Abdul Wahab, Shahrul Hisham Zainal Ariffin

**Affiliations:** 1 Centre for Research and Graduate Studies, University of Cyberjaya, 63000, Cyberjaya, Selangor Malaysia; 2 Department of Food Sciences, Faculty of Science and Technology, Universiti Kebangsaan Malaysia, 43600 Bangi, Selangor Malaysia; 3 Department of Earth Sciences and Environment, Faculty of Science and Technology, Universiti Kebangsaan Malaysia, 43600 Bangi, Selangor Malaysia; 4 School of Biology, Faculty of Applied Sciences, Universiti Teknologi MARA, 40450 Shah Alam, Selangor Malaysia; 5 Department of Biological Sciences and Biotechnology, Faculty of Science and Technology, Universiti Kebangsaan Malaysia, 43600 Bangi, Selangor Malaysia; 6 Malaysia Genome Institute, National Institutes of Biotechnology Malaysia, Ministry of Science, Technology and Innovation (MOSTI), Jalan Bangi, 43000 Kajang, Selangor Malaysia; 7 Centre of Family Dental Health, Faculty of Dentistry, Universiti Kebangsaan Malaysia, Jalan Raja Muda Abdul Aziz, 50300 Kuala Lumpur, Wilayah Persekutuan Kuala Lumpur Malaysia

**Keywords:** Biotechnology, Experimental organisms

## Abstract

In Malaysia, *Piper sarmentosum* or ‘kaduk’ is commonly used in traditional medicines. However, its biological effects including in vivo embryonic toxicity and tissue regenerative properties are relatively unknown. The purpose of this study was to determine zebrafish (*Danio rerio*) embryo toxicities and caudal fin tissue regeneration in the presence of *P. sarmentosum* aqueous extracts. The phytochemical components and antioxidant activity of the extract were studied using GC–MS analysis and DPPH assay, respectively. Embryo toxicity tests involving survival, heartbeat, and morphological analyses were conducted to determine *P. sarmentosum* extract toxicity (0–60 µg/mL); concentrations of 0–400 µg/mL of the extract were used to study tissue regeneration in the zebrafish caudal fin. The extract contained several phytochemicals with antioxidant activity and exhibited DPPH scavenging activity (IC_50_ = 50.56 mg/mL). Embryo toxicity assays showed that a concentration of 60 μg/mL showed the highest rates of lethality regardless of exposure time. Slower embryogenesis was observed at 40 µg/mL, with non-viable embryos first detected at 50 µg/mL. Extracts showed significant differences (*p* < 0.01) for tissue regeneration at all concentrations when compared to non-treated samples. In conclusion, *Piper sarmentosum* extracts accelerated tissue regeneration, and extract concentrations at 60 µg/mL showed the highest toxicity levels for embryo viability.

## Introduction

Natural products have been frequently used as dietary supplements for health promotion and for culinary uses due to their herbaceous scents and flavours. *Piper sarmentosum* is one particular natural product with several medicinal effects including anti-cancer and fracture healing properties^1, 2^. *P. sarmentosum* is a medicinal plant found in tropical countries, such as Indonesia, the Philippines, and Malaysia. The plant is widely consumed in the Asean region for its medicinal benefits. Aqueous extracts from *P. sarmentosum* have been found to produce anti-cancer effects on human liver cancer and Chang’s cell lines^2, 3^. However, further study on Chang’s cell lines which originally derived from normal liver has found this cell is indistinguishable from HeLa (human cervical cancer) by STR PCR^4^. *P. sarmentosum* extracts have been reported to produce aid in healing fractures; such extracts contain flavonoid compounds that reduce bone loss and increase bone strength in ovariectomised rats^1^. *P. sarmentosum* aqueous extracts also reduce oxidative stress and increase antioxidant defence mechanisms in diabetic rats^5^. Oral ingestion of *P. sarmentosum* aqueous extract of up to 2000 mg/kg/day do not exhibit sub-acute toxicities in Sprague Dawley rats^6^. However, the in vivo toxicity effects of *P. sarmentosum* extracts on zebrafish embryos (vertebrate model), and its regenerative potential in tissues regeneration at early and adult stages have not yet been reported.

The zebrafish has emerged as a valuable and cost effective model organism to study tissue regeneration and embryo development in vertebrates due to its small size, the ability to regenerate many tissues and organs over short periods of time, and easy maintenance^7, 8^. Zebrafish was used in this study as a vertebrate model to investigate the effects of *P. sarmentosum* extracts during embryo developmental stages and adult tissue regeneration. Early zebrafish embryo stages are sensitive when subjected to external stimuli including toxicants, chemicals, and mechanical stresses^9^. This development stage can be exploited as a suitable vertebrate model for toxicity assessments. Zebrafish also have short life cycles, multiple offspring production, and transparent developmental stages which provide advantages for toxicity models^10^. Zebrafish are widely used to study the effects of chemicals, such as pesticides, nanoparticles, and various organic pollutants in the environment^11–13^. In addition, the zebrafish genome is 80–85% similar to humans^14, 15^, which supports its suitability as an in vivo model for toxicity and tissue regenerative studies and applicability for human diseases treatments such as osteoarthritis.

In this study, the phytochemicals of *P. sarmentosum* aqueous extract were identified using gas chromatography-mass spectrometry (GC–MS) analysis, and antioxidant activity of the extract was evaluated by the 2, 2-diphenyl-1-picrylhydrazyl (DPPH) assay. Embryo toxicity tests were conducted to determine the toxicity of aqueous extracts from *P. sarmentosum*. These tests are often used to determine the toxicity of chemicals that may affect both environmental health and human health^16, 17^. We also determined the safety levels or dosage of *P. sarmentosum* extracts that effected embryo development and tissue regeneration. Toxicity tests and tissue regeneration capacities of *P. sarmentosum* aqueous extracts were performed to investigate the safety of this extract towards zebrafish at two different stages of life, embryo and adult, as a model for humans. Assessing the toxicity of plant extracts on the development stage of the embryo is important since some plants are widely consumed and even commercialized without information on their toxicology profile. The generated results can be used as references for further human safety consumption.

## Results

### Identification of phytochemical components

**Figure 1:**
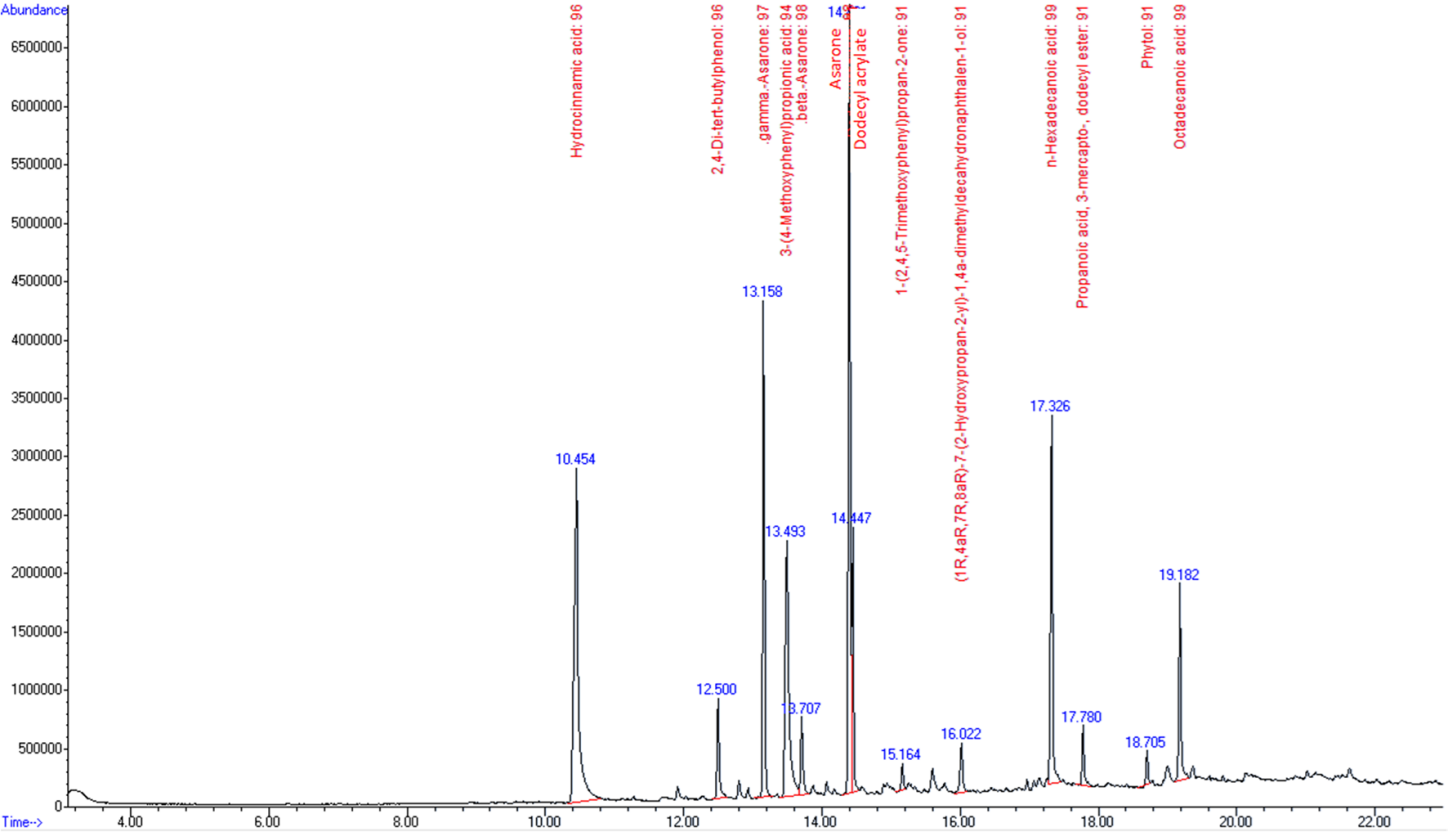
GC–MS chromatograms of *P. sarmentosum* aqueous extract.

GC–MS analysis of the aqueous extract of *P. sarmentosum* (Fig. 1) showed 13 peaks which indicated the presence of 13 phytochemical constituents. On comparison of the mass spectra of the constituents with the NIST library, the 13 phytocompounds were characterized and identified (Table 1). The various phytochemicals which contribute to the medicinal activities of the plant were shown in Table 1. The first compound identified was hydrocinnamic acid, with the shortest retention time (10.45 min). Octadecanoid acid was the final identified compound with a retention time of 19.18 min. The most prevailing compounds identified with the highest peak area in percentage were hydrocinnamic acid (16.49%) and asarone (16.49%), followed by 3-(4-methoxyphenyl) propionic acid (10.9%) and gamma-asarone (9.89%). Among the 13 compounds identified, seven compounds had been reported to exhibit antioxidant activity (Table 1).

**Table 1:** Phytochemical compounds and bioactivities identified in *P. sarmentosum* aqueous extract.

Number	Retention time (minutes)	Name of the compound	Area Percentage (%)	Activity
1	10.4542	Hydrocinnamic acid	16.4865	Antioxidant^28^
2	12.5001	2,4-Di-tert-butylphenol	2.5803	Antioxidant^29^
3	13.1587	gamma-Asarone	9.8914	Larvicidal^53^
4	13.4909	3-(4-Methoxyphenyl)propionic acid	10.9005	No activity reported
5	13.7066	beta-Asarone	2.2622	Antioxidant^30^
6	14.4002	Asarone	16.4926	Antioxidant^31^
7	14.4468	Dodecyl acrylate	5.4597	No activity reported
8	15.1638	1-(2,4,5-Trimethoxyphenyl)propan-2-one	0.9606	No activity reported
9	16.0206	(1R,4aR,7R,8aR)-7-(2-Hydroxypropan-2-yl)-1,4a-dimethyldecahydronaphthalen-1-ol	1.5666	Antipasmodic^54^
10	17.3262	n-Hexadecanoic acid	9.3402	Antioxidant^32^
11	17.7808	Oxalic acid, propyl tetradecyl ester	1.5682	No activity reported
12	18.7076	Phytol	0.8218	Antioxidant^33^
13	19.1797	Octadecanoic acid	4.5081	Antioxidant^34^

### Antioxidant activity of *P. sarmentosum* extract

**Figure 2:**
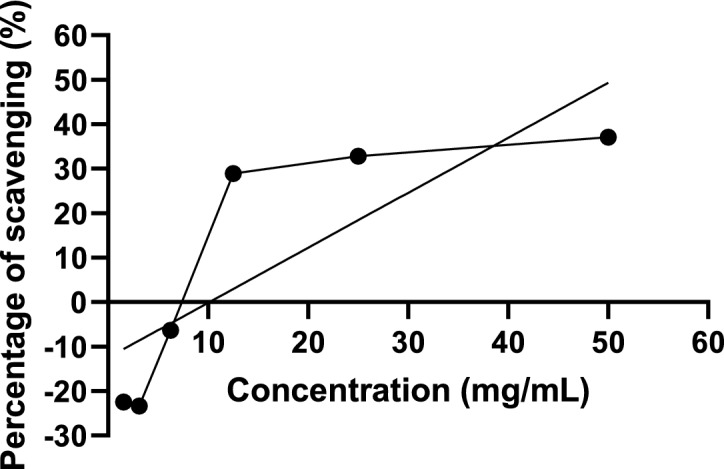
DPPH radical scavenging activity of *P. sarmentosum* aqueous extract (n = 5).

The antioxidant activity of different concentrations of *P. sarmentosum* extract was determined. The free radical scavenging effect is increased in proportion to concentration; this indicates that the extract possesses antioxidant activity. The IC_50_ value for *P. sarmentosum* extract was determined using linear regression and was found to be 50.56 mg/mL. Figure 2 shows the antioxidant activity of DPPH radical.

### Embryos treated with *P. sarmentosum* extracts; survival analyses

**Figure 3:**
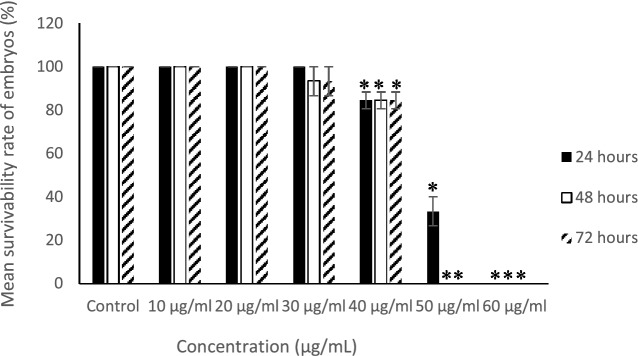
Survival rate at 24, 48 and 72 h (n = 3; 15 embryos/replicate).

Survival analyses of zebrafish embryos showed no significant differences (*p* > 0.01) in embryo survival rates for treatments < 30 µg/mL. However, survival rates were decreased by approximately 80% when embryos were exposed to 40 µg/mL extracts. Survival rates decreased further (~ 30%) when embryos were treated with 50 µg/mL for 24 h, and no embryos survived after 48–72 h exposure at this concentration (Fig. 3). Finally, the 60 µg/mL treatment killed all embryos at 24, 48, and 72 hpf (Fig. 3). In contrast, the control group (0 µg/mL) showed 100% embryo survival after 24, 48, and 72 hpf. These results were based on mean data from 15 embryos. These data suggest that embryo survival decreased with increasing extract concentrations in a time-dependent manner. The results also indicate that at 60 μg/mL, the extract was lethal for embryo survival.

### Embryo heartbeat analysis

**Figure 4:**
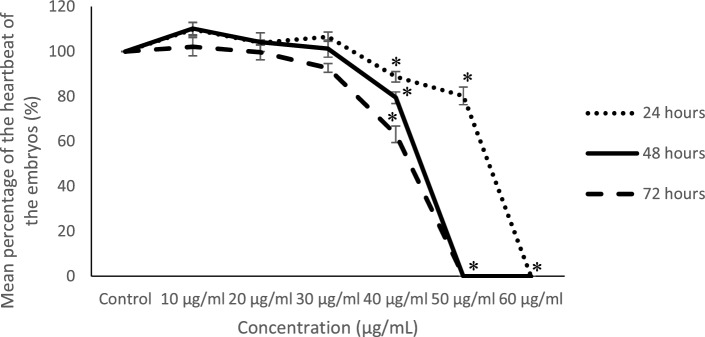
Percentage of embryos’ heartbeat at 24, 48 and 72 hpf (n = 3; 15 embryos/replicate).

Using unpaired t-tests, no significant differences (*p* > 0.01) in mean heartbeat percentages were observed for 10 µg/mL, 20 µg/mL, and 30 µg/mL treatments when compared to untreated control embryos (Fig. 4). However, the mean heartbeat percentage at 40 µg/mL was found to be significantly lower (*p* < 0.01) at 24, 48, and 72 hpf when compared to control embryos. In contrast, embryos treated with 50 and 60 µg/mL extracts showed no heartbeats, as no embryos survived these concentrations. While heartbeats could still be detected when embryos were treated with a concentration of 50 µg/mL, toxic effects were still exhibited at 24 hpf, represented by significantly lower (*p* < 0.01) heartbeat compared to control. These results suggest that the concentration was toxic but non-lethal to the embryos.

### Embryo morphology analyses

**Figure 5:**
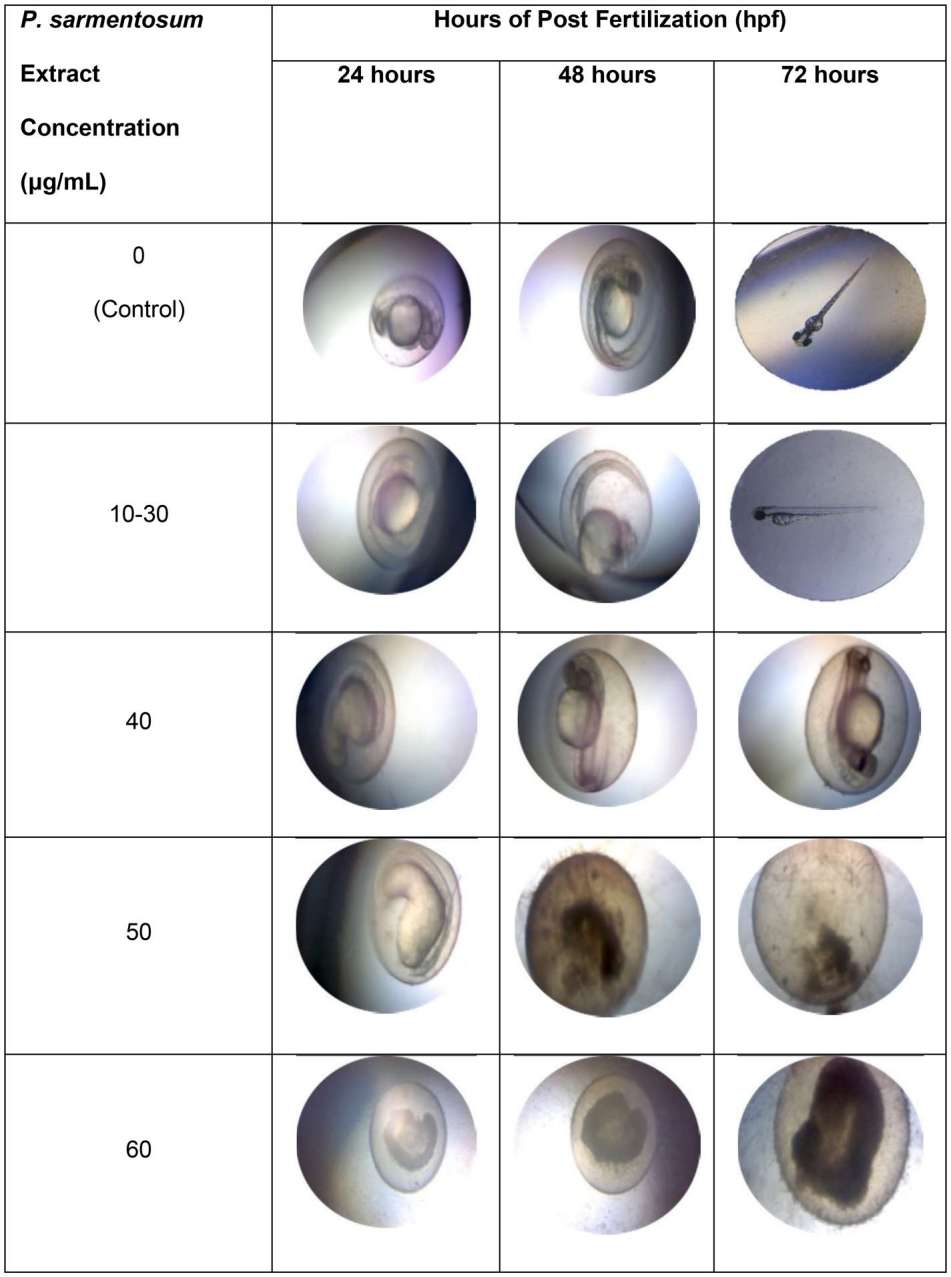
Embryo morphology at 24, 48 and 72 h at different concentration extract (n = 15).

Embryo morphological analyses showed similar growth levels for the control group and the 10–30 µg/mL groups (Fig. 5). However, embryo development slowed when treated with 40 μg/mL extract. Embryos were unable to survive when treated with 50 μg/mL and 60 μg/mL at 24–72 hpf (Fig. 5). Extracts at 40 µg/mL exhibited decreased rates of embryo development, while 50 μg/mL and 60 μg/mL extracts exhibited toxic and harmful effects in embryos as presented by the non-viable morphology, including embryo coagulation and undeveloped organs such as the spine, tail, and heart. Therefore, extract concentrations at 40 μg/mL or higher were toxic to embryo development, with lethal effects observed at 50–60 µg/mL as evidenced by all 15 non-viable embryos after 24 hpf (Fig. 5).

### Zebrafish caudal fin tissue regeneration

**Figure 6:**
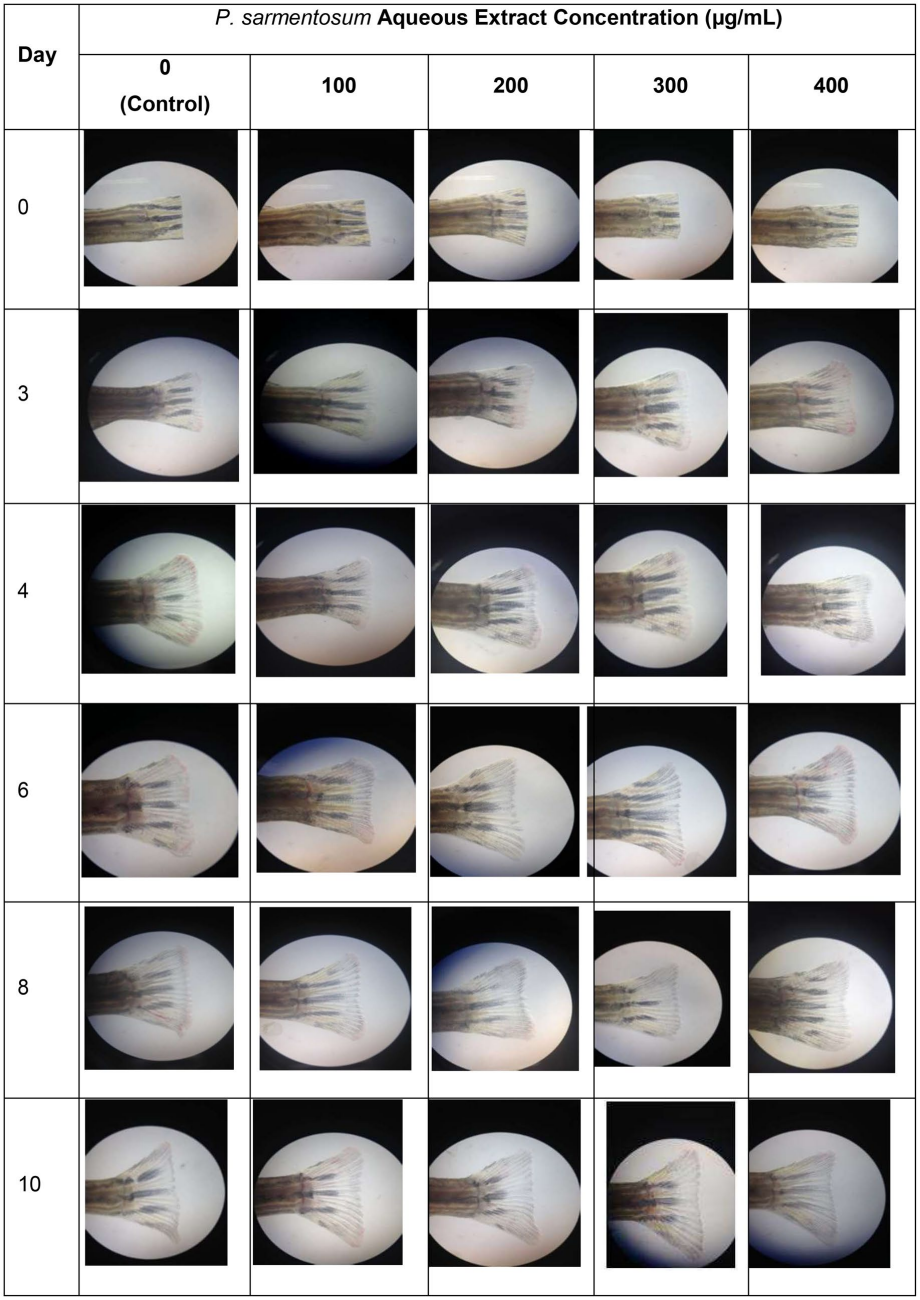
Caudal fin regeneration for 10 days in various concentrations of *P. sarmentosum* aqueous extract (n = 9).

**Figure 7:**
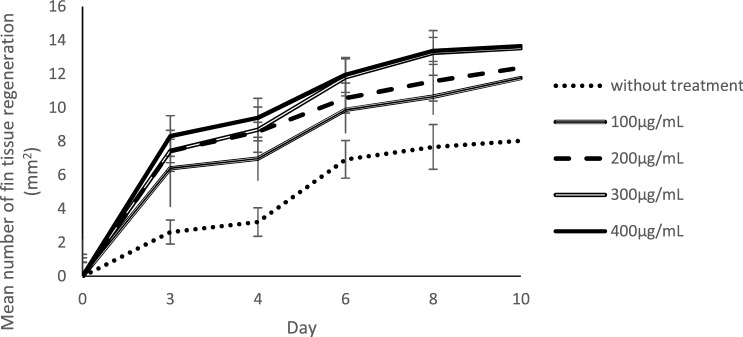
Tissue regeneration of Zebrafish caudal fin (each test represented by 6 male and 3 female zebrafish; n = 9).

Images of caudal fins after amputation and treatment with extracts are shown in Fig. 6. The results showed that caudal fins, treated with extracts, were significantly regenerated (*p* < 0.01) when compared to the control group. The 400 µg/mL treatment had the highest and fastest regenerative rate (Fig. 7). These data indicated that the regeneration of caudal fin tissue was accelerated when *P. sarmentosum* aqueous extract concentrations were increased.

### Comparisons between male and female caudal fin tissue regeneration

Observations on amputated caudal fin regeneration in male and female zebrafish, after 0–400 µg/mL extract treatments, were performed for 10 days (Table 2). Caudal fin regeneration rates after 10 days for males at 0 µg/mL, 100 µg/mL, 200 µg/mL, 300 µg/mL and 400 µg/mL extracts were 0.77 ± 0.1 mm^2^/day, 1.17 ± 0.13 mm^2^/day, 1.21 ± 0.13 mm^2^/day, 1.38 ± 0.15 mm^2^/day, and 1.33 ± 0.06 mm^2^/day, respectively. Similarly, caudal fin regeneration rates for females were 0.88 ± 0.18 mm^2^/day, 1.19 ± 0.06 mm^2^/day, 1.28 ± 0.10 mm^2^/day, 1.29 ± 0.08 mm^2^/day, and 1.43 ± 0.09 mm^2^/day, respectively. The pattern for caudal fin regeneration in female zebrafish is slightly different compared to male zebrafish, where increment of regeneration rates with increased concentration was observed after 10 days. For male zebrafish, the regeneration rate was observed to be decreased with higher concentration including 400 µg/mL. At day 10, the caudal fin regeneration process has found to be diminished in male zebrafish treated with 400 µg/mL *P. sarmentosum* aqueous extract. However, unpaired t-test analyses on caudal fin regeneration areas showed no significant differences (*p* > 0.01) between males and females at all concentrations: 100 µg/mL (*p* = 0.373), 200 µg/mL (*p* = 0.222), 300 µg/mL (*p* = 0.176) and 400 µg/mL (*p* = 0.051).

**Table 2:** Regeneration area of Zebrafish caudal fin treated with *P. sarmentosum* aqueous extract.

Concentration of *P. sarmentosum* (µg/mL)	Mean for caudal fin regeneration area of Zebrafish on respective day after amputation (mm^2^)									
	Day 3		Day 4		Day 6		Day 8		Day 10	
	Male	Female	Male	Female	Male	Female	Male	Female	Male	Female
0	2.8 ± 0.98	2.3 ± 0.22	3.5 ± 0.67	2.6 ± 0.24	6.6 ± 0.70	7.7 ± 0.62	7.4 ± 1.13	8.1 ± 1.12	7.7 ± 0.97	8.8 ± 1.83
100	7.3 ± 2.13	4.6 ± 0.58	7.9 ± 2.25	5.1 ± 0.41	10.2 ± 0.92	9.2 ± 1.95	10.8 ± 1.51	10.3 ± 1.37	11.7 ± 1.29	11.9 ± 0.61
200	7.3 ± 0.80	7.7 ± 1.04	8.7 ± 0.76	8.4 ± 0.60	10.6 ± 0.56	10.6 ± 0.66	11.2 ± 0.69	12.3 ± 0.88	12.1 ± 1.26	12.8 ± 1.03
300	7.6 ± 1.13	7.1 ± 1.17	9.0 ± 0.94	8.1 ± 1.73	12.4 ± 0.03	10.6 ± 1.23	13.7 ± 1.10	12.4 ± 0.30	13.8 ± 1.48	12.9 ± 0.80
400	8.1 ± 1.53	8.9 ± 0.57	9.0 ± 1.32	10.1 ± 0.52	11.8 ± 1.42	12.2 ± 0.42	13.2 ± 1.21	13.7 ± 0.63	13.3 ± 0.59	14.3 ± 0.91

*Six male (n = 6) and three females (n = 3) were used for each concentration.

## Discussion

Our embryo survival analyses showed that embryo survivability decreased with increasing extract concentrations in a time-dependent manner, where higher concentrations and longer exposure times affected embryo survival. The percentage of surviving embryos was drastically decreased after 24 h exposure to 50 µg/mL extract, with no embryo surviving after 48 and 72 h. This showed that high extract concentrations and longer exposure times exerted pressures on zebrafish embryo development. During early developmental stages, the weakened protective layer (chorion) around the zebrafish embryo will be easily affected by influx of external solutes, including the aqueous extract of *Piper sarmentosum*^18^. Prolonged exposure to the extract can lead to increase in its accumulation, until a concentration that can induce toxicity in the embryos is reached^19^. Similar toxicity patterns were also observed for *Tinospora cordifolia* extracts, where zebrafish embryo survival was also dependent on extract doses and exposure times. Increase of dose and time of exposure led to increased mortality of zebrafish embryos exposed to *Tinospora cordifolia*^20^. Similarly, exposure of *Piper sarmentosum* extract at higher doses and for longer periods also decreased the survivability of zebrafish embryos in our study.

In this study, embryo heartbeat rate analyses showed decreases in embryo heartbeats after exposure to 40 µg/mL extracts at 24, 48, and 72 hpf when compared to control embryos. Heartbeat decrement rates were also reported in zebrafish embryos exposed to zearalenone, naproxen, hexabromocyclododecane, and caffeine^21, 22^. Toxic effects of these compounds on embryo development were also investigated using heartbeat analysis. The heartbeat rates in our zebrafish embryos were affected by 40 µg/mL *P. sarmentosum* extracts, and no heartbeat was detected at 50–60 µg/mL. This data suggests that exposure to *P. sarmentosum* extract at these concentrations may thus affect embryo cardiac function.

A normal zebrafish embryo can be assessed by morphological features: a straight spine, normal body shape, round yolk sac, and pigmentation on the body and eyes. In contrast, abnormal development is demonstrated by a bent spine, an enlarged yolk sac, pericardial oedema, and a slow heartbeat^23^. Exposure to toxicants can also lead to egg coagulation and undeveloped organs such as the spine, tail, and heart^23^. In this study, embryo development declined along with decreases in heartbeat rates when embryos were exposed to 40 µg/mL of extract. In addition, exposure to higher extract concentrations (50 µg/mL and 60 µg/mL) caused embryo coagulation. Overall, *P. sarmentosum* extracts (≥ 40 µg/mL) were found to be toxic to the embryos and may affect cardiac function, as evidenced by morphological abnormalities and reductions in heartbeat, together with decreased embryo survival.

The early development stages of zebrafish embryos are the most sensitive phases in terms of external stimuli^9^. Supplementation of high concentrations of *P. sarmentosum* aqueous extracts may be toxic to embryos, but not adults. A toxicity study using the same extract in a different animal model demonstrated that ingestion of extract up to 2000 mg/kg/day did not cause toxicity in 7- or 8-week-old Sprague Dawley rats^6^. This observation suggested that extract safety levels were dependent on the developmental stage of the organism.

In this study, extracts were also used to study tissue regeneration of *P. sarmentosum* extracts through the analysis of amputated caudal fin regeneration. *P. sarmentosum* extracts have shown the ability to accelerate fracture healing in osteoporotic mice^24^. The extracts used in this study are soluble in aqueous solutions, which may be a significant advantage in terms of potential medical applications in humans. The aqueous extract also reduced bone resorption by decreasing cortical levels in rats^25^. The ability to regenerate the zebrafish caudal fin could be due to the antioxidant properties of *P. sarmentosum* aqueous extracts, which may accelerate bone healing^26^. Moreover, these antioxidant properties can help endogenous antioxidant defence systems to protect bone from osteoporosis in a rat model^27^. The exhibited antioxidant activity could be associated with hydrocinnamic acid, 2,4-di-tert-butylphenol, beta-asarone, asarone, n-hexadecenoic acid, phytol, or octadecanoic acid, which are abundantly present in the *P. sarmentosum* aqueous extracts^28–34^. The most abundant compound, hydrocinnamic acid, is a derivative of cinnamic acid, a compound that exhibits strong antioxidant activity due to the presence of a CH=CH–COOH (prop-2-enoic acid) moiety^35–38^. Antioxidant activities of *P. sarmentosum* extracts identified through DPPH assays have also been reported in other studies^39, 40^. The DPPH radical model was also applied in our study to evaluate the free radical scavenging activity of *P. sarmentosum* aqueous extract. Use of the DPPH assay to observe antioxidant activity is facilitated by the ability of antioxidants to donate hydrogen that leads to the decrease in absorbance of DPPH^41^. Therefore, it is postulated that *P. sarmentosum* extracts possess antioxidant properties that promote faster tissue regeneration in our study.

Gender differences could also influence the tissue regeneration of the caudal fin. Male zebrafish showed poor regenerative capacities of pectoral fins when compared to female zebrafish^42^. However, in our study, observations at day 10 showed no significant differences (*p* > 0.01) between males and females in terms of caudal fin regeneration, despite small variations in the increment pattern between the genders. This suggests that *P. sarmentosum* extracts have the potential to accelerate zebrafish caudal fin regeneration, regardless of gender. Regeneration in females was found to continuously increase in a concentration-dependent manner, while fin regeneration in male zebrafish had exhibited decreased rates of regeneration at a concentration of 400 µg/mL. However, larger sample sizes and balanced samples between genders are needed for better understanding of this finding.

The caudal fin in zebrafish is an excellent model for appendage regeneration and bone repair^43^. Caudal fin regeneration occurs within one to two weeks after amputation, and appears to have an infinite capacity to regenerate in terms of original size, tissue architecture, and function^44^. Skeletal tissue is one of the main components of the fin and is composed of several segmented bony rays, produced by osteoblasts, i.e., bone secreting cells^45^. Several bone studies in rats have shown that the administration of *P. sarmentosum* extract increases bone strength and enhances fracture healing processes; antioxidant properties in leaf extracts showed high levels of flavonoid compounds which reduce bone loss^1, 24, 46^. Our study demonstrated that treatment with 100–400 µg/mL extract accelerated caudal fin regeneration after amputation, when compared with untreated controls. This suggests that *P. sarmentosum* extracts contribute to regenerating caudal fins.

After amputation, three main regeneration phases are activated. The first phase is wound healing, which occurs 0–18 h post-amputation (hpa). In this phase, epithelial cells cover the wound by forming a wound epidermis and secreting factors such as Fgf20a and Activin-βA to induce the next phase of the regeneration process. Wound healing is followed by blastema formation (18–48 hpa)^47^. In the final phase, a regenerative outgrowth occurs 48 h to 10 days after amputation. During this phase, patterning and differentiation occur to restore tissue architecture and caudal fin function^48^. In our study, observations at day 10 after amputation showed that tissue architecture was restored, and regeneration of the caudal fin was increased with administration of *P. sarmentosum* aqueous extract after amputation. In another study, the regeneration of caudal fin was observed to be higher in zebrafish fed with aqueous extracts of different plant, *Boletus qriseipurpureus*^49^. In our study, extracts administered at a concentration as low as 100 µg/mL were able to accelerate regeneration at day three by at least two-fold higher compared to untreated controls. Higher concentrations of *P. sarmentosum* extract also affected the rate of caudal fin regeneration. These data suggest that this extract is a potential inducer of tissue regeneration.

Zebrafish embryos, showed decreased survival, reduced heartbeats, and abnormalities in embryo morphology when exposed to *P. sarmentosum* aqueous extracts at a concentration of 40 µg/mL. However**,** zebrafish caudal fin regeneration rates were accelerated when concentrations of *P. sarmentosum* aqueous extracts were increased. This could be due to the observed antioxidant activity of the extracts, which demonstrated an IC_50_ value at 50.56 mg/mL; this result supported the crude extract analysis which showed that six of 12 identified compounds were reported as antioxidant. The ability of the extracts used in this study to be soluble in aqueous solution represents a potential clinical advantage in human applications. These data suggest that *P. sarmentosum* aqueous extracts induce detrimental effects in zebrafish embryo development, but induce accelerated tissue regeneration in the adult.

## Methods

### Sampling and plant aqueous extraction

Fresh leaves from *P. sarmentosum* were collected from the Forest Research Institute of Malaysia (FRIM), Kuala Lumpur, Malaysia. They were identified by a botanist from the Faculty of Applied Science, Universiti Teknologi MARA (UiTM). Plant extraction was conducted at the Faculty of Science and Technology, Universiti Kebangsaan Malaysia (UKM). Leaves were prepared by cleaning and drying in a 50 °C oven for one week. The dried leaves were then ground to a powder using pestle and mortar at room temperature and stored in an air tight container in the dark at 4 °C prior to extraction.

Approximately 100 g powdered samples were boiled in distilled water (1:2 w/v) for 90 min. Mixtures were centrifuged at 6,000 rpm using Mikro 22 R centrifuge machine (Hettich, Germany) for 10 min to generate a supernatant. The centrifugation process was repeated until no pellet was observed. The supernatant was transferred to a new tube and preserved by freeze-drying using a freeze dryer Alpha 1–2 LD Plus (Christ, Germany). Once dried, the powder was stored in the dark at 4 °C until required. The dried powder was weighed and dissolved in distilled water for further analysis.

Plant extract concentrations at 0–60 µg/mL were prepared for embryo toxicity analyses. For caudal fin regeneration analyses, concentrations of 0–400 µg/mL were prepared due to sensitivity of the embryos towards the extract and that no significant difference in tissue regeneration using lower extracts concentrations was observed compared to control. *P. sarmentosum* aqueous extracts were dissolved in sterile distilled water to generate stock concentrations for analyses.

### Compound Identification via GC–MS

A total of 50 mg *P. sarmentosum* aqueous extract was diluted with 1 mL sterile ultrapure water. Diluent was mixed with 1 mL methanol and fractionation was carried out using 0.5 mL dichloromethane. The aqueous dichloromethane fraction was isolated prior to gas chromatography-mass spectrometry (GC–MS) analysis. GC–MS was performed using an Agilent 7,890 gas chromatograph coupled to an Agilent 5,975 quadrupole mass detector (Agilent Technologies, Santa Clara, USA) equipped with a HP-5MS capillary column (30 m × 250 µm inner diameter × 0.25 µm film). The oven temperature was initially maintained at 40 °C for 2 min, followed by a two-step temperature increase to 175 °C at a rate of 5 °C/min, then to 250 °C at 90 °C/min with helium carrier gas flow rate set at 1 mL per min. The temperature of the ion source and transfer line was set at 220 °C and 280 °C, respectively, and electron impact mass spectra was recorded at 70 eV ionization energy. One µL of extract was injected into GC injection port at a temperature of 250 °C using a split mode of 1:50. The volatile compounds were identified by mass spectra comparison using MSD Chemstation Enhanced Data Analysis Software (E.02.02.1431 version, Agilent Technologies) software and National Institute of Standards and Technology library database (NIST 14). The relative amount of the individual component was expressed as a percentage relative to the total peak areas of all identified volatiles.

### Determination of antioxidant activity using DPPH assay

Radical scavenging activities of *P. sarmentosum* aqueous extract and ascorbic acid (standard) were measured using the DPPH assay^50^, where the ability of the extract to donate a hydrogen atom was determined by the decolorization of an ethanol solution of DPPH. Fresh ascorbic acid (standard) and *P. sarmentosum* aqueous extracts were prepared at 50 mg/mL using ultrapure water. Samples were serially diluted with 0.1 M sodium acetate pH 6.0, with concentration ranged from 1.5625 mg/mL to 50 mg/mL at final volume of 50 µL per well. Equal amounts of 5 mM DPPH were added to each sample and standard followed by incubation in the dark for 25 min. After 25 min, activity was measured at 530 nM, where the DPPH purple colour in ethanol solution faded to yellow in the presence of antioxidants.

### Zebrafish embryo toxicity

Zebrafish embryos were supplied by the Biochemistry Department, Faculty of Biotechnology and Biomolecule Science, UPM. Zebrafish eggs were collected and selected under a light microscope at 1 h post fertilisation (hpf). Fertilised eggs were cleaned using sterile distilled water and incubated at 28 °C in E3 medium (5 mM NaCl, 0.17 mM KCl, 0.33 mM CaCl_2_, 0.33 mM MgSO_4_ and 0.1% (w/v) methylene blue).

Maintenance and breeding procedures for zebrafish were performed in accordance with the Organisation for Economic Co-operation and Development (OECD) guidelines^51^. The breeding process began with the separation of male and female zebrafish into different tanks for six days. During this period, they were fed with high protein pellets. A high protein diet promotes health and increases embryo production. During this time, all zebrafish were exposed to light for 14 h, followed by 10 h of darkness. On the sixth day, male and female zebrafish in a ratio of 3:1 were placed in the dark for 10 h, followed by exposure to 3 h of light to induce mating^52^. After mating, zebrafish embryos were collected and rinsed three times in sterile distilled water. After six hpf, five embryos were transferred to each well of 96-well plates in the presence of 10–60 µg/mL of *P. sarmentosum* aqueous extract in triplicate (15 embryos/replicate)*.* Untreated embryos (0 µg/mL extract) were used as a control group.

Embryos were observed under a light microscope (magnification 40x) (Olympus, Japan). The survivability, heartbeat, and morphological changes were recorded at 24, 48, and 72 hpf. Since the heart is the first internal organ to form and function during zebrafish development, at approximately 24–48 hpf, heartbeats can reflect toxicological impact^23^. The percentage of surviving embryos and associated heartbeats, along with embryonic morphological analyses, were used to assess the impact of *P. sarmentosum* aqueous extracts on embryos.

### Caudal fin regenerative capacity

For regenerative capacity studies, six males and three female zebrafish, aged two months old, were used for each extract concentration. Zebrafish were anesthetised using 0.01% tricaine (Sigma-Aldrich), and amputations of the caudal fin were performed using a sterile razor blade. Translucent sections of caudal fin regeneration tissues can be observed by the naked eye. Thin epidermal layers were formed after amputation. These layers were important for the formation of blastema, which affects regeneration of the caudal fin^43^. During regenerative outgrowth, blastema which identified as mesenchymal stem cells will proliferate and differentiate to replace amputated structures. All procedures were performed according to animal guidelines described in Muniandy et al.^49^.

The caudal fin of the zebrafish was amputated approximately 5 mm proximal to the first branch point of the lepidotrichia. Concentrations of *P. sarmentosum* aqueous extracts at 100, 200, 300, and 400 µg per gram of fish body weight in 5 µL, were administered to zebrafish by oral gavage, twice daily for 10 days. Untreated male and female zebrafish were used as a control group and received 5 µL distilled water. Caudal fin regeneration was measured and recorded at days 0, 3, 4, 6, 8, and 10 using microscopy. Images were analysed on Image-J software to quantify regeneration areas. Caudal fin regeneration rate was calculated as the regeneration of fin areas (mm^2^) grown over the number of days.

### Statistical analyses

Data were expressed as the mean ± standard deviation from three independent experiments, except for embryo morphology analyses which were performed using 15 embryos. Statistically significant differences (*p* < 0.01) between treatments on embryo survival and heartbeats were analysed using unpaired t-tests, whereas paired t-tests were used for tissue regeneration analyses.

### Ethical approval

This research was carried out according to the ethical and legal requirements of Universiti Kebangsaan Malaysia, Animal Ethical Committee (UKMAEC). This permission allowed us to use zebrafish while abiding to legal and ethical guidelines. Zebrafish protocols were performed humanely throughout this research. They were anaesthetised prior to amputation and oral gavage. All described experimental protocols involving zebrafish were designed and performed according to the animal ethics guidelines approved by the UKMAEC with approval reference number FST/2019/SHAHRUL HISHAM/25-SEPT./1032-OCT.-2019-SEPT.-2020.

## Data Availability

The datasets generated during and/or analysed during the current study are available in the Figshare repository, https://doi.org/10.6084/m9.figshare.12110616.
